# Early accelerated senescence of circulating endothelial progenitor cells in premature coronary artery disease patients in a developing country - a case control study

**DOI:** 10.1186/1471-2261-13-104

**Published:** 2013-11-19

**Authors:** Kranthi Vemparala, Ambuj Roy, Vinay Kumar Bahl, Dorairaj Prabhakaran, Neera Nath, Subrata Sinha, Pradipta Nandi, Ravindra Mohan Pandey, Kolli Srinath Reddy, Ajay Manhapra, Ramakrishnan Lakshmy

**Affiliations:** 1 Department of Cardiac Biochemistry, All India Institute of Medical Sciences, New Delhi, India; 2 Department of Cardiology, All India Institute of Medical Sciences, New Delhi, India; 3 Center for Chronic Disease Control and CARRS COE, Public Health Foundation of India, New Delhi, India; 4 Department of Biochemistry, All India Institute of Medical Sciences, New Delhi, India; 5 National Brain Research Center, Manesar, Haryana, India; 6 Department of Biostatistics, All India Institute of Medical Sciences, New Delhi, India; 7 Public Health Foundation of India, New Delhi, India; 8 Department of Medicine, University of Virginia School of Medicine, Charlottesville, VA, USA

**Keywords:** Premature coronary artery disease, Endothelial progenitor cells, Senescence, Telomere length, Telomerase activity

## Abstract

**Background:**

The decreased number and senescence of circulating endothelial progenitor cells (EPCs) are considered markers of vascular senescence associated with aging, atherosclerosis, and coronary artery disease (CAD) in elderly. In this study, we explore the role of vascular senescence in premature CAD (PCAD) in a developing country by comparing the numerical status and senescence of circulating EPCs in PCAD patients to controls.

**Methods:**

EPCs were measured by flow cytometry in 57 patients with angiographically documented CAD, and 57 controls without evidence of CAD, recruited from random patients ≤ 50 years of age at All India Institute of Medical Sciences. EPC senescence as determined by telomere length (EPC-TL) and telomerase activity (EPC-TA) was studied by real time polymerase chain reaction (q PCR) and PCR– ELISA respectively.

**Result:**

The number of EPCs (0.18% Vs. 0.039% of total WBCs, p < 0.0001), and EPC-TL (3.83 Vs. 5.10 kb/genome, p = 0.009) were markedly lower in PCAD patients compared to controls. These differences persisted after adjustment for age, sex, BMI, smoking and medications. EPC-TA was reduced in PCAD patients, but was statistically significant only after adjustment for confounding factors (1.81 Vs. 2.20 IU/cell, unadjusted p = 0.057, adjusted p = 0.044).

**Conclusions:**

We observed an association between increased vascular cell senescence with PCAD in a sample of young patients from India. This suggests that early accelerated vascular cell senescence may play an important mechanistic role in CAD epidemic in developing countries like India where PCAD burden is markedly higher compared to developed countries.

## Background

While coronary vascular disease (CVD) and coronary artery disease (CAD) occurs predominantly among older individuals in developed countries, it occurs at earlier ages in developing countries. The proportion of CVD deaths below 70 years of age in 1990 was only 22.8% in the established market economies, whereas it was 52.2% in India [1]. It is unclear why communities where CAD is emerging as a threat have a high burden of premature coronary artery diseases (PCAD) [2].

The development of CAD among elderly has been associated with the progression of senescence in almost all cellular elements of the vascular system [3,4]. Endothelial dysfunction is presumed to play a crucial role in the development of atherosclerosis and coronary artery disease spectrum including PCAD [5]. Circulating endothelial progenitor cells (EPCs) mobilized from the bone marrow by the vascular system provide an endogenous repair mechanism for endothelial cell injury [6], and may therefore have an important role in the pathophysiology of CAD progression. Numerical decline and functional impairment of circulating EPCs has been associated with aging and senescence of vascular system, and more pronounced numerical and functional decline of EPCs have been associated with CAD among elderly [3,7-9]. Reduced telomere length is considered a marker of senescence in various cells including those in vascular system. Reduced telomere length in EPCs (EPC-TL) is also associated with CAD among elderly [3,7-9]. In addition, increased EPC senescence has been shown to be associated with higher risk for cardiovascular events in individuals [10], and risk factors and clinical conditions like diabetes and hypertension are known to have detrimental effect on numerical and functional levels of EPCs in CAD patients [11-15]. Based on demonstration of extensive association of vascular cell senescence with atherosclerosis and CAD, and evidence of shared biochemical pathways between vascular aging and atherosclerosis, others have suggested that CAD among elderly can be viewed as accelerated aging [4,16]. However, the role of accelerated vascular aging in the pathogenesis of PCAD has not been explored before in detail especially in the setting of a developing country like India where the burden of PCAD is very high. In this study we explore the role of numerical and functional decline of EPCs, and telomere shortening of EPCs, markers of vascular senescence in PCAD patients from India.

## Methods

### Subjects

The Ethics Committee of the All India Institute of Medical Sciences (New Delhi, India), approved the protocol and written informed consent was obtained from all subjects prior to the study. A total of 57 subjects, age ≤ 50 years of either sex, with evidence of CAD as confirmed by the presence of either > 50% stenosis in the left main coronary artery or > 70% stenosis in other arteries on coronary angiography were recruited for the study group. Fifty-seven age matched (± 5 years) non-diabetic subjects of both genders without known heart disease and either having a normal coronary angiogram or without demonstrable ischemia on stress testing were recruited as controls from random patients visiting Cardiology department of All India Institute of Medical Sciences (AIIMS), New Delhi, India from 2008 to 2010. Subjects with any form of cancer, renal disease, rheumatoid arthritis, pneumonia, nephropathy and cerebrovascular disease were excluded. 30 ml fasting venous blood sample was collected in EDTA tubes. 200 μl of the whole blood was used for EPC enumeration. Plasma was separated from 3 ml of blood for biochemical analysis. Rest of the blood was used for EPC isolation for measurement of telomere length and telomerase activity. Insulin, hsCRP (high sensitive C-reactive protein), and homocysteine were analyzed by ELISA using kits from Mercodia (Sweden), Biochek (CA) and Diazyme (CA) respectively. Glucose was measured by glucose oxidase, total cholesterol by CHOD-PAP, and triglycerides by GPO-PAP methods using kits from RANDOX (Crumlin, UK). High density lipoprotein (HDL) cholesterol was measured by precipitation method using RANDOX kits. Insulin resistance was calculated using Homeostatic Model assessment (HOMA). Low Density Lipoprotein (LDL) was calculated using friedwald’s formula.

### EPC enumeration

EPCs were quantified in blood by flow cytometry. Briefly 200 μl of peripheral blood was treated with FcR blocking reagent (MiltenyiBiotec, Germany) and incubated with Fluorescein isothiocyanate (FITC) labeled CD34 (BD Biosciences, USA) and R-phycoerythrin (PE) labeled VEGFR2 antibodies (R&D systems, USA). Labeled cells were lysed with Fluorescence-activated cell sorting (FACS) lysis buffer (BD Biosciences, USA) and fixed. Baseline parameters, compensation settings and gates were set using unstained controls and isotype controls. EPCs were identified as CD34+/KDR + dual positive cells in the upper right quadrant region. A total of 500,000 events were recorded in each unstained and stained sample. The numbers of CD34+/KDR + cells were expressed as a percentage of total WBC.

### EPC isolation

Mononuclear cells (MNC) were isolated from blood samples by HISTOPAQUE®-1077 (Sigma chemicals, India) density gradient centrifugation according to manufacturer’s protocol. EPCs were isolated using a two step protocol of Magnetic Activated Cell Sorting (MACS) using CD 34 multisort kit (MiltenyiBiotec, Germany) and indirect labeling procedure with PE labeled VEGFR2 (R&D systems, India) and anti-PE micro beads (Miltenyi Biotec, Germany). Briefly, MNC were treated with FcR blocking reagent (Miltenyi Biotec, Germany) and incubated with CD34 multisort beads and PE labeled VEGFR2. The CD34 labeled cells were separated through positive selection and incubated with anti-PE microbeads and cells which were dual positive for CD34/VEGFR2 collected, counted and aliquoted for telomere length and telomerase activity assays.

### Measurement of EPC Telomere length (EPC-TL)

EPC-TL was measured by real time PCR method as described by Cawthon et al. [17] Callaghan et al. [18] and N Callaghan and Fenech [19] with minor modifications.

### Measurement of EPC Telomerase activity (EPC-TA)

EPC Telomerase activity (EPC-TA) was measured by a combination of telomeric repeat amplification protocol (TRAP) and photometric enzyme immunoassay according to manufacturers protocol (Roche Diagnostics, India).

### Statistical methods

Statistical analysis was performed using STATA statistical software (version 9.0 for windows). Fifty patients and 50 controls were needed for 90% power and α of 0.05. We elected to recruit additional subjects within a stipulated time and a ceiling of 60. Normality of the sampling distribution of each variable was tested using Kolmogorov-Smirnov test for normality. The distributions of EPC number, EPC-TL, and EPC-TA were not normal and therefore log transformed for analysis. Continuous variables like age, height, weight, BMI, systolic blood pressure, diastolic blood pressure, medication dose, left ventricular ejection fraction (LVEF)% and biochemical estimates were expressed as Mean ± SD (Standard Deviation) and categorical variables were expressed as numbers and percentage. Log transformed variables were expressed as geometric mean with 95% confidence intervals (95% CI). Baseline characteristics and biochemical estimates of cases and controls were compared using unpaired student’s *t* test. Categorical variables that were not normally distributed were analyzed using Wilcoxon rank sum test. Student’s *t* test was used to compare the means of EPC number, EPC-TL, and EPC-TA in cases and controls. Linear regression analysis was employed to adjust for confounding variables. Bivariate and partial correlations were computed for assessing correlations between EPC number/EPC senescence and biochemical parameters. Confounding variables taken for adjustment included age, sex, BMI, smoking and medications. Statistical significance was assumed if P value was less than or equal to 0.05.

## Results

The baseline characteristics are detailed in Tables 1 and 2. The proportion of female patients were low in both PCAD (1) or control group (4), and family history of CAD was more often present in PCAD group. Use of statins, ACE-inhibitors, β blockers and aspirin was significantly higher in PCAD. PCAD group had lower mean total cholesterol, LDL, HDL and triglycerides, possibly reflecting higher statin use, but had significantly higher homocysteine levels compared to controls.

**Table 1:** Baseline characteristics of Subjects

**Parameter**	**PCAD (N = 57)**	**Controls (N = 57)**	**P value**
**Age (yrs)**	43.1 ± 6.36	39.8 ± 6.13	0.125
**Height (cm)**	165.8 ± 5.63	168.9 ± 6.99	0.008
**Weight (kg)**	69.1 ± 11.25	69.2 ± 12.30	0.959
**BMI (kg/m**^**2**^**)**	24.8 ± 3.96	24.2 ± 3.91	0.472
**Family history for CAD (n) (%)**	18 (31.6)	6 (10.5)	0.006
**Family history for hypertension (n) (%N)**	2 (3.5)	6 (10.5)	0.142
**SBP (mmHg)**	127.9 ± 11.88	127.46 ± 14.87	0.855
**DBP (mmHg)**	83.4 ± 6.82	81.8 ± 8.95	0.290
**Statins, n (%)**	36 (63.0)	7 (12.0)	<0.001
**ACE – Inhibitors, n (%)**	21 (37.0)	6 (10.5)	<0.001
**ß – blockers, n (%)**	28 (49.0)	2 (3.5)	<0.001
**Aspirin, n (%)**	41 (72.0)	10 (17.5)	<0.001
**PPAR-γ agonists n (%)**	1 (2.0)	0 (0.0)	0.229
**LVEF% (mean ± SD)**	44.6 ± 1.77	60.40 ± 1.56	0.001
**Diabetes n (%)**	9 (15.8)	--	
**Hypertension, n (%)**	13 (22.8)	14 (24.6)	0.785
**Smoking, n (%)**	24 (42.1)	14 (24.6)	0.047
**Alcohol, n (%)**	11 (19.3)	16 (28.1)	0.083

**Table 2:** Biochemical characteristics of subjects

	**PCAD**	**Controls**	**P value**
	**Means ± SD**	**Means ± SD**	
	**(N = 57)**	**(N = 57)**	
**Total cholesterol (mg/dl)**	128.5 ± 32.7	171.1 ± 36.0	<0.001
**Triglycerides (mg/dl)**	112.6 ± 59.2	175.5 ± 108.5	<0.001
**HDL (mg/dl)**	35.3 ± 7.1	40.8 ± 5.5	<0.001
**VLDL (mg/dl)**	22.7 ± 11.9	34.4 ± 20.0	<0.001
**LDL (mg/dl)**	72.7 ± 30.5	96.2 ± 25.3	<0.001
**LDL/HDL ratio**	2.13 ± 0.96	2.4 ± 0.84	0.076
**Glucose (mg/dl)**	100.3 ± 44.3	96.4 ± 15.5	0.477
**Insulin (mU/L)**	8.9 ± 6.1	11.5 ± 10.7	0.074
**Insulin resistance**	2.31 ± 2.0	2.89 ± 3.2	0.199
**CRP (mg/dl)**	3.01 ± 3.4	3.5 ± 3.03	0.316
**Homocysteine (μmol/L)**	18.23 ± 11.99	13.65 ± 9.90	0.038

### Circulating EPC number and senescence

**Figure 1:**
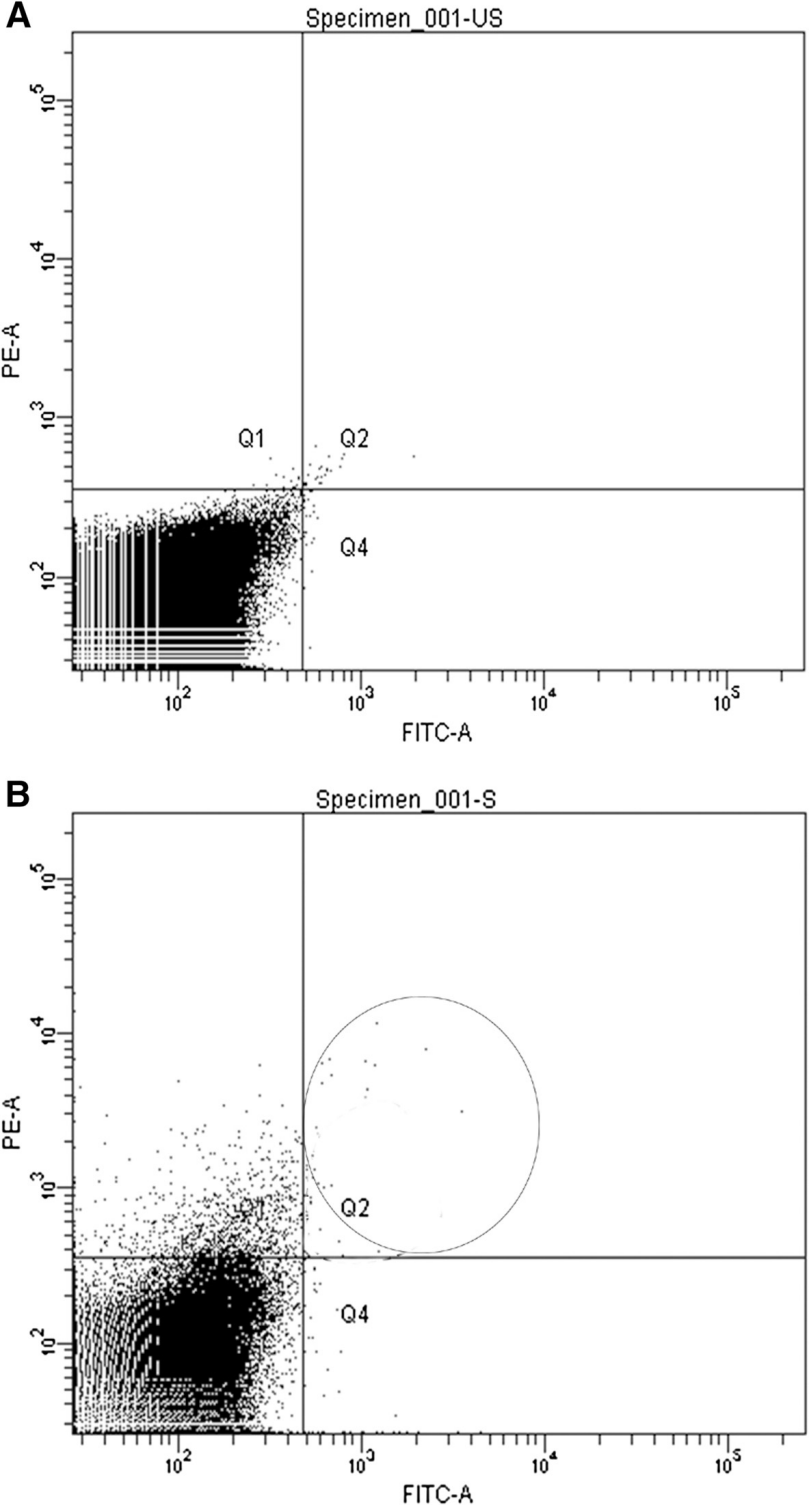
**Unstained (A) and stained (B) quadrangle plot of CD34-Fluorescence isothiocyanate in forward scatter and versus VEGRF2 (KDR)-phycoerythrin in the side scatter in one representative patient sample.** The upper right quadrant shows the dual stained EPCs.

Figure 1A and 1B shows the flow cytometry analysis of circulating EPCs. Figure 1B shows the stained EPCs. As shown in Table 3 the mean percent of EPCs were significantly lower in PCAD patients compared to controls, and this persisted after adjustment for confounding variables. The mean EPC-TL was also markedly lower in PCAD patients compared to controls and the difference remained significant after adjustment. The mean relative EPC-TA was lower in PCAD patients as compared to controls, but the difference was statistically significant (P = 0.044) only after adjusting for confounding variables. Additionally adjusting for family history of diabetes did not change these associations. The EPC numbers were lower in smokers as compared to non smokers (0.022% vs. 0.014%) but the difference was not statistically significant (p = 0.127).

**Table 3:** Number and senescence of circulating endothelial progenitor cells in premature CAD patients compared to normal controls

**Parameter**	**PCAD patients**	**Controls**	**P value**	Adjusted P value^*^
**EPC number (% of total WBC)**	**0.018 (0.013-0.023)**	**0.039 (0.031-0.043)**	**<0.001**	**0.001**
**EPC telomere length (kb/genome)**	**3.83 (3.20-4.50)**	**5.10 (4.26-6.10)**	**0.009**	**0.050**
**EPC telomerase activity (IU/cell)**	**1.81 (1.30-2.40)**	**2.20 (1.50-3.20)**	**0.057**	**0.044**

*Adjusted for age, sex, BMI, smoking and medication.

### Correlation of biochemical parameters with circulating EPC levels and EPC senescence

In controls, EPC number positively correlated with total cholesterol before and after adjusting for age, sex, BMI and smoking (Unadjusted Pearson r = 0.231, P = 0.021, Adjusted Pearson r = 0.218, P = 0.033). However the association was lost when adjusted for medications. HDL levels were positively correlated with EPC number (Unadjusted Pearson r = 0.284, P = 0.004, adjusted r = 0.241, P = 0.018). In PCAD patients EPC numbers negatively correlated with triglyceride levels (unadjusted Pearson r = -0.280, p = 0.049 and adjusted Pearson r = -0.380, P = 0.010). EPC-TL was also correlated with triglycerides (Unadjusted Pearson r = -0.326, P = 0.014 and adjusted r = -0.289, P = 0.038). EPC-TL positively correlated with EPC-TA (Pearson r = 0.409, P = 0.002).

## Discussion

We observed markedly lower numbers of circulating EPCs in young patients with angiographically documented CAD from India as compared to controls without CAD. In addition, the EPCs from PCAD patients also showed a marked decrease in telomere length and telomerase activity. The difference remained even after adjusting for all the confounding variables (age, sex, BMI, use of medication and smoking). Our findings suggest that PCAD patients in India have accelerated vascular senescence compared to those without CAD. EPC number and telomere length also negatively correlated with serum triglyceride levels in patients.

The results reported in the present study are in agreement with other studies reporting a reduced EPC number in older CAD patients. Vasa et al. [20] reported lower EPC (CD34/KDR positive) numbers and function in CAD patients with a mean age of 62 years. Eizawa et al. has also reported a similar reduction in EPC (CD34+ cells) number in CAD patients [21]. Aging associated numerical and functional decline in EPCs in CAD patients has been attributed to exhaustion of stem/progenitor cells in the bone marrow due to chronic vascular injury, reduced mobilization, diminished migratory and adhesion capacity of EPCs and deregulation of EPC differentiation [22,23], and is thought to render the elderly more prone to endothelial dysfunction and cardiovascular disease [24]. Results of our study suggest that low EPC levels may be responsible for endothelial dysfunction and atherosclerosis in PCAD patients as well.

A shorter EPC-TL in PCAD patients reported in the present study points towards accelerated senescence of EPCs resulting in lower EPC number, suggestive of compromised repair of dysfunctional endothelium. EPC telomere shortening and decreased EPC telomerase activity has been reported in older patients with stable and unstable CAD [25,26]. Kushner et al. reported age related decline of EPC-TL in healthy men from a developed nation, starting after the age of 55 years, and EPC-TL was 20% shorter in older as compared to middle aged men and young suggesting EPC telomere shortening may be responsible for age related endothelial dysfunction [23]. In our study, the PCAD patients with a mean age of 43 years show a similar decline in TL (25% reduction) compared to controls of similar age, suggesting an earlier onset of EPC senescence in them. Increased oxidative stress associated with metabolic derangements has been suggested as an explanation for the shorter EPC telomeres and EPC lower telomerase activity [25], and this could be applicable to younger patients too. We did not measure markers of oxidative stress in our study. The negative correlation between triglycerides and EPC number and senescence on our study would be relevant as Indians are known to consume high carbohydrate diet, and have higher triglyceride levels. The PCAD patients showed lower triglyceride levels possibly due to statin use [26]. Other authors have also reported an association between EPC and triglycerides [27]. Triglyceride rich lipoproteins have been shown to increase senescence of endothelial progenitor cells via oxidative stress [28]. Since the subjects in this study had a mean age of 43, age is unlikely to be the culprit for low EPC count and higher level of EPC senescence in PCAD patients. This EPC senescence is probably driven by multiple factors including increased oxidative stress due to smoking, and atherogenic lipoprotein phenotype characterized by high triglycerides and low HDL.

Recent studies have shown that diet may have significant influence on EPC-TL and EPC-TA [29]. As with cardiac risk factors, non-pharmacologic intervention like exercise [30,31], and diet modification [29,32] can positively influence vascular aging along with medications like statins [33]. Statins are known to promote EPC mobilization, proliferation, migration, adhesion, and differentiation, and reduce senescence. We did not find any significant correlation between dose of statins and EPC number and EPC senescence in our study possibly because most of our subjects were on low dose of statins.

Though prematurely speculative, this study with the context of prior evidence from developed countries provides interesting insights into the mechanisms of premature CVD in Indians. Autopsy studies from United States have shown that coronary atherosclerosis have markedly declined from 77% prevalence in 1950s to 8.5% in 2011, a period of sharp decline in premature CAD death and risk factor prevalence in United States [34], suggesting that the high CAD burden among middle aged adults in 1940s and 1950s in United States (as seen now in countries like India and China) was probably driven by “reservoir” of atherosclerosis in the young population, and the abundance of noxious risk factors [35]. Aging associated atherosclerosis can be considered as accelerated vascular aging probably influenced by additional pathological burden induced by various other noxious environments like smoking, dyslipidemia, and hypertension [16], and atherosclerosis occurring at younger age could be due to early accelerated aging of the vascular wall [4]. Consistent with this view, our data suggests that senescence of vascular system may be happening early in an accelerated fashion in PCAD patients from developing countries. Nilsson et al. has suggested that the early vascular aging including telomere anomalies that lead to atherosclerosis is probably a reflection of more generalized early biological aging in susceptible individuals, and this susceptibility is partly driven stresses of early life like fetal health and early childhood adverse growth patterns [36], a situation still common in countries like India. Putting all of this together, it is tempting to speculate that as survival rates at young ages in developing countries increase and the high exposure to fetal and early life stresses persist, the “reservoir” of susceptible people with early vascular aging (and general biological aging) in developing country like India can be very high. The exponentially increasing prevalence of atherogenic risk factors at young adulthood in developing countries [37] may be further accelerating the vascular senescence in the population, especially among the already susceptible ones. This complex interplay between early-accelerated vascular senescence biology and the environmental factors like urbanization, nutrition, and ecology of living environments is probably a crucial driver of the epidemic of premature CAD in developing countries.

We have not excluded patients who are on statin therapy, which influence EPC number. We have however adjusted for the effect of statins and other medications in statistical analysis. Further, we have not looked at the functional and mobilization capacity of EPCs in premature CAD patients, possibly of greater importance than absolute EPC number and needs to be addressed in future studies. Unlike many prior studies EPC-TL and EPC-TA in our study was measured directly in EPC isolated from individuals, and not from EPC cultures, and this more realistically mimic in-vivo conditions. Moreover the specific role of EPCs in development of atherosclerosis and CAD is still being clarified.

## Conclusion

Significantly lower EPCs in premature CAD patients in the present study suggest impaired repair mechanism predisposing to endothelial dysfunction at younger ages. Association of EPC senescence with triglycerides, which is part of the atherogenic South Asian phenotype, is suggestive of a possible role of classical risk factors in regulating EPC number in this population. A shorter EPC telomere length and a reduced telomerase activity, in the young CAD patients in the present study, points to an accelerated senescence of EPCs in vivo, resulting in lower circulating EPCs. Together with prior data, our study suggests that early-accelerated vascular senescence may be a crucial driver of premature CAD in developing countries. The study also suggests that EPC telomere length could be used as an early marker for detecting impaired repair mechanism predisposing to endothelial dysfunction in premature CAD patients. The pliability of vascular aging to control of risk factors, and the possible involvement of early life factors in the generation of early accelerated vascular senescence/aging suggests that national policies focusing simultaneously on early life enhancement and chronic risk factor reduction maybe likely to curtail the burden of premature CAD in developing countries.

## Abbreviations

BMI: Body mass index; CVD: Coronary vascular disease; CAD: Coronary artery disease; EPC’s: Endothelial progenitor cells; EPC-TL: Endothelial progenitor cell telomere length; EPC-TA: Endothelial progenitor cell telomerase activity; EDTA: Ethylene diamine tetra acetic acid; FACS: Fluorescence-activated cell sorting; FITC: Fluorescein isothiocyanate; hsCRP: High sensitive C-reactive protein; HDLc: High density lipoprotein cholesterol; HOMA: Homeostatic Model assessment; LDLc: Low density lipoprotein cholesterol; LVEF: Left ventricular ejection fraction; MACS: Magnetic activated cell sorting; MNC: Mononuclear cells; PCAD: Premature coronary artery disease; PCR-ELISA: Polymerase chain reaction enzyme linked immunoabsorbent assay; PE: R-phycoerythrin; qPCR: Real time polymerase chain reaction; TRAP: Telomeric repeat amplification protocol; WBC’s: White blood cells.

## Competing interests

The authors declare that they have no competing interests.

## Authors’ contributions

KV carried out the experiments, performed the statistical analysis and drafted the manuscript. LR conceived the study, participated in its design and coordination and helped to draft the manuscript. DP helped in designing the study and gave intellectual inputs for writing the manuscript. VKB, PN and AR helped in recruitment of subjects. SS and NN helped in experimental design. RMP helped in performing the statistical analysis. AM gave intellectual inputs and edited the manuscript. KSR provided intellectual inputs for study design. All authors read and approved the final manuscript.

## Pre-publication history

The pre-publication history for this paper can be accessed here:

http://www.biomedcentral.com/1471-2261/13/104/prepub
